# Supplementary material for: FastqPuri: high-performance preprocessing of RNA-seq data
Source: BMC Bioinformatics. 2019 May 3;20:226. doi: 10.1186/s12859-019-2799-0 (PMC6500068; doi:10.1186/s12859-019-2799-0)
Supplement: Supplementary file 2 — Archive of FastqPuri. Archive containing all files needed to install and run FastqPuri v1.0.6. Date stamp March 22, 2019. (GZ 47,819 kb) [file 12859_2019_2799_MOESM2_ESM.gz › FastqPuri-1.0.6/html/structstatsinfo.html]

FastqPuri: statsinfo Struct Reference


FastqPuri

Public Attributes |
List of all members

statsinfo Struct Reference

stores info needed to create the summary graphs
More...

`#include <stats_info.h>`

|  |  |
| --- | --- |
| Public Attributes | |
| int | read\_len |
|  | |
| int | ntiles |
|  | |
| int | nQ |
|  | |
| int | minQ |
|  | |
| int | tile\_pos |
|  | |
| int | nreads |
|  | |
| int | reads\_wN |
|  | |
| int | sz\_lowQ\_ACGT\_tile |
|  | |
| int | sz\_ACGT\_tile |
|  | |
| int | sz\_reads\_MlowQ |
|  | |
| int | sz\_QPosTile\_table |
|  | |
| int | sz\_ACGT\_pos |
|  | |
| int \* | tile\_tags |
|  | |
| int \* | lane\_tags |
|  | |
| int \* | qual\_tags |
|  | |
| uint64\_t \* | lowQ\_ACGT\_tile |
|  | |
| uint64\_t \* | ACGT\_tile |
|  | |
| uint64\_t \* | reads\_MlowQ |
|  | |
| uint64\_t \* | QPosTile\_table |
|  | |
| uint64\_t \* | ACGT\_pos |
|  | |

## Detailed Description

stores info needed to create the summary graphs

## Member Data Documentation

## ◆ ACGT\_pos

uint64\_t\* statsinfo::ACGT\_pos

# A, C, G, T, N per position

## ◆ ACGT\_tile

uint64\_t\* statsinfo::ACGT\_tile

# A, C, G, T, N per tile, to compute the fraction of lowQuality bases per tile and per nucleotide.

## ◆ lane\_tags

int\* statsinfo::lane\_tags

Names of the existing tiles

## ◆ lowQ\_ACGT\_tile

uint64\_t\* statsinfo::lowQ\_ACGT\_tile

# low Quality A, C, G, T, N per tile

## ◆ minQ

int statsinfo::minQ

Minimum quality threshold

## ◆ nQ

int statsinfo::nQ

# possible quality values

## ◆ nreads

int statsinfo::nreads

# reads read till current position.

## ◆ ntiles

int statsinfo::ntiles

# tiles

## ◆ QPosTile\_table

uint64\_t\* statsinfo::QPosTile\_table

# bases of a given quality per tile.

## ◆ qual\_tags

int\* statsinfo::qual\_tags

Names of the existing qualities

## ◆ read\_len

int statsinfo::read\_len

Maximum length of a read

## ◆ reads\_MlowQ

uint64\_t\* statsinfo::reads\_MlowQ

# reads with M(position) lowQuality bases.

## ◆ reads\_wN

int statsinfo::reads\_wN

# reads with N's found till current position

## ◆ sz\_ACGT\_pos

int statsinfo::sz\_ACGT\_pos

ACGT\_pos size = read\_len \* N\_ACGT

## ◆ sz\_ACGT\_tile

int statsinfo::sz\_ACGT\_tile

ACGT\_tile size = ntiles \* NACGT

## ◆ sz\_lowQ\_ACGT\_tile

int statsinfo::sz\_lowQ\_ACGT\_tile

lowQ\_ACGT\_tile size = ntiles \* N\_ACGT

## ◆ sz\_QPosTile\_table

int statsinfo::sz\_QPosTile\_table

QposTile\_Table size = ntiles \* nQ \* read\_len

## ◆ sz\_reads\_MlowQ

int statsinfo::sz\_reads\_MlowQ

reads\_MlowQ size = read\_len + 1

## ◆ tile\_pos

int statsinfo::tile\_pos

current tile position

## ◆ tile\_tags

int\* statsinfo::tile\_tags

Names of the existing tiles

---

The documentation for this struct was generated from the following file:

- include/stats\_info.h


---

Generated on Mon Mar 19 2018 23:42:01 for FastqPuri by  

 1.8.14
